# What Healthcare Professionals Think of “Nutrition & Diet” Apps: An International Survey

**DOI:** 10.3390/nu12082214

**Published:** 2020-07-24

**Authors:** Maria F. Vasiloglou, Stergios Christodoulidis, Emilie Reber, Thomai Stathopoulou, Ya Lu, Zeno Stanga, Stavroula Mougiakakou

**Affiliations:** 1 ARTORG Center for Biomedical Engineering Research, University of Bern, Murtenstrasse 50, 3008 Bern, Switzerland; maria.vasiloglou@artorg.unibe.ch (M.F.V.); stergios.christodoulidis@artorg.unibe.ch (S.C.); thomai.stathopoulou@artorg.unibe.ch (T.S.); ya.lu@artorg.unibe.ch (Y.L.);; 2 Institute Gustave Roussy, Inserm Unit, U981 Paris, France; 3 Department of Diabetes, Endocrinology, Nutritional Medicine and Metabolism, Inselspital, Bern University Hospital, University of Bern, 3010 Bern, Switzerland; emilie.reber@insel.ch (E.R.); Zeno.Stanga@insel.ch (Z.S.); 4 Department of Emergency Medicine, Inselspital, Bern University Hospital, University of Bern, 3010 Bern, Switzerland

**Keywords:** mHealth, mobile applications, smartphone, dietary assessment, survey, healthcare professionals

## Abstract

Accurate dietary assessment is crucial for both the prevention and treatment of nutrition-related diseases. Since mobile-based dietary assessment solutions are promising, we sought to examine the acceptability of “Nutrition and Diet” (ND) apps by Healthcare Professionals (HCP), explore their preferences on apps’ features and identify predictors of acceptance. A 23 question survey was developed by an interdisciplinary team and pilot-tested. The survey was completed by 1001 HCP from 73 countries and 6 continents. The HCP (dietitians: 833, doctors: 75, nurses: 62, other: 31/females: 847, males: 150, neither: 4) had a mean age (SD) of 34.4 (10.2) years and mean job experience in years (SD): 7.7 (8.2). There were 45.5% who have recommended ND apps to their clients/patients. Of those who have not yet recommended an app, 22.5% do not know of their existence. Important criteria for selecting an app were ease of use (87.1%), apps being free of charge (72.6%) and validated (69%). Significant barriers were the use of inaccurate food composition database (52%), lack of local food composition database support (48.2%) and tech-savviness (43.3%). Although the adoption of smartphones is growing and mobile health research is advancing, there is room for improvement in the recommendation of ND apps by HCP.

## 1. Introduction

The prevention and treatment of diet-related diseases is a pressing issue of global concern. With an accurate assessment of dietary consumption and eating behaviour it should be possible to prevent and treat many nutrition-related diseases and to maintain balanced eating patterns. As part of the dietary assessment process, healthcare professionals seek to assess people’s dietary patterns to then suggest corrective actions. Understanding and recording what, and when people eat, fundamentally depends on accurate dietary assessment tools, and mobile health (mHealth) technology holds great promise as an approach [1]. It is estimated that more than five billion people worldwide own mobile devices, and more than half of these are smartphones [2]. Mobile-assisted approaches for dietary monitoring may reduce costs related to execution time and involvement of experts and more importantly measurement errors that conventional methods of dietary assessment, such as food diaries, are prone to [3]. To this end, some “Nutrition and Diet” (ND) apps use images of dishes (before and/or after food consumption) to estimate energy and nutrient content. Two different approaches can be employed: (i) Manual: the user needs to guide the system to select the type of food or/and the portion size, (ii) Automatic or semi-automatic: the type of food and/or the portion size are estimated by the system using Artificial Intelligence (AI) and image analysis techniques and minimal or no input from the user [4,5,6,7,8]. The food items are then identified, labelled, segmented and reconstructed in three dimensions. The findings are then used in combination with a food composition database, and the food image is translated into quantities of nutrients and energy estimation [6,7,8]. These approaches have several advantages, including real-time dietary feedback and less dependency on the respondent′s memory [9]. However, limitations may include under-reporting due to poor image quality or if the user misses, forgets, or deliberately chooses not to record certain meals [10].

If healthcare professionals (HCPs) are to successfully integrate technology into medical management they must be capable of using data from health apps to support diagnosis and to propose advice for treatment [11,12]. HCPs who recommend apps can use them as supplementary tools to broaden their daily practice, engage patients, enhance care, and possibly contribute to the reduction in health care costs [13]. Additionally, patients living with diseases such as diabetes or obesity may use them for the self-monitoring of their diet and/or physical activity [14]. Although frequently employed in dietetic practice, smartphone health apps and other mHealth technologies are not often used for behavioural modification, nor are they an integral part of the nutritional care process [15].

A number of studies on how HCPs use and regard diet apps for dietary assessment and monitoring have already been conducted. A survey that was conducted among sports dietitians found that nearly 30% of them use apps in their practice and find them useful for monitoring and assessment [16]. In another study, clinicians mention that ND apps may improve patient outcomes when compared to conventional methods [14]. Other researchers mentioned that dietitians prefer apps designed for patient-specific needs and also apps which can be integrated into their work systems [15]. Moreover, in a Canadian study, it was found that even if dietitians were enthusiastic about ND apps, they expressed a number of concerns related to their use in daily practice, such as lack of credibility, accuracy, or content [17]. However, the aforementioned studies are mainly focused on Australia, the UK, New Zealand, and the USA [14,15,16,17,18].

The factors that influence the use of health apps by dietitians and their patients must be identified. The essential issue is how to align and improve the design of interventions and the implementation of the apps, in order to assist and optimise the processes of nutrition care and clinical practice. Since HCPs are the key actors of dietary monitoring and assessment we needed to figure out their expectations. We seek answers on questions such as whether apps are recommended or not by the HCPs, whether they find them useful and also gather opinions on what is missing, what is needed and what can be improved. Despite the vast number of ND apps in the marketplace, to our best knowledge there are no global studies on the perspectives, preferences and recommendations of HCPs. We have therefore conducted an international survey to understand the opinions of HCPs with respect to apps for dietary monitoring and assessment. Our study aims to (i) identify the acceptability of ND apps by HCPs worldwide; (ii) explore the preferences of HCPs in relation to app features, reliability and functionality; and (iii) explore predictors of acceptance of ND apps.

## 2. Materials and Methods 

**Figure 1:**
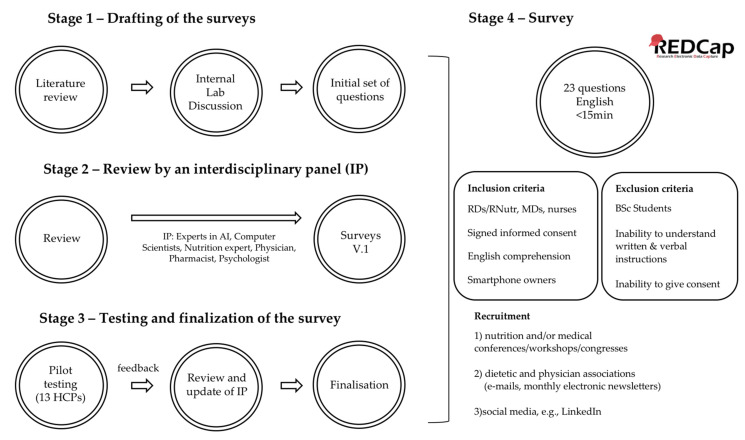
Overview of the study design.

An observational, multinational, interdisciplinary survey was performed consisting of closed multiple choice questions, Yes/No questions and some open questions. Where necessary, technical terms were explained in a footnote. The survey was developed in an iterative, three-stage process, which is described in the following sections and shown in Figure 1.

**Stage 1—Drafting of the survey:** The preliminary questions were developed by the members of the “AI in Health and Nutrition Laboratory” of the University of Bern after reviewing literature on surveys conducted using health/diet apps and also on the innovative technologies that have been proposed for dietary assessment. Through internal discussions we concluded a first draft of the survey for HCPs (40 main questions; 11 of which involved demographics, 18 questions that were taken either unchanged or slightly modified by other surveys with similar objectives [14,15,16,17] and another 11 were developed by our team).

**Stage 2—Review by an interdisciplinary panel:** The draft survey was reviewed by an interdisciplinary panel (experts in AI, computer scientists, nutrition expert, physician, pharmacist, psychologist). The panel provided suggestions about adding or removing questions and also about the phrasing of the questions. The first panel agreed to 30 changes and the creation of 36 revised main questions. After some days of the survey completion by each HCP and after receiving their comments on it, a new questionnaire was sent to each of them to verify their given answers. We also asked them to provide more comments on the structure, content, readability and sequence of questions, duration and length, as well as if they had any additional comments. The final survey included 30 main questions. During the optimisation process, some questions were rephrased to make them easier to understand. For some questions, the spectrum of possible answers was extended either by splitting them into more questions or by including new multiple-choice selections.

**Stage 3—Testing and finalisation of the surveys:** To establish the validity of the content, we tested the draft questions using pilot responders, in order to find out whether the survey was simple, understandable, clear, easy to complete, concise and user-friendly, as well as to ensure its validity and acceptability. For this reason, we invited 18 HCPs selected for their scientific expertise: 8 physicians; 8 dietitians/nutritionists; 1 nurse and 1 pharmacist. An e-mail was sent to the potential participants, providing them with the necessary information for their task and asking them for their agreement. If they agreed to participate, a link to the survey was provided to the testing responders. The responders were asked to provide feedback on the structure (user-friendliness of the design, ease of navigation), content (comprehensiveness, adequate coverage of the topic, expected questions that were not asked, questions that might affect response rate), readability and flow of questions (sequence, unfamiliar terminology used, language simplicity, grammar or syntax mistakes, complex questions), and duration (time needed, length) [19,20,21]. A total of 13 HCPs (72% response rate) accepted the invitation to pilot-test the survey.

**Stage 4—Survey:** In response to the comments of the pilot responders of the survey, the interdisciplinary panel revised the draft questions. The final survey included a total of 23 questions, 22 of which had to be answered before proceeding to the next section and one was optional, asking for the e-mail address of the participants. The survey was created and managed using REDCap (Research Electronic Data Capture) electronic data capture tool. REDCap is a secure, Web-based app designed to support data capture for research studies and provides interfaces in different devices such as laptops or smartphones. The survey was available in English. Respondents had the possibility of reviewing all their answers throughout the survey, but responses were locked upon clicking the “Submit” button. The survey required 5–15 min to complete, depending on the paths taken through the survey, but no time limit was set. The possibility of saving incomplete surveys and continuing later was also offered.

The inclusion criteria were signed informed consent before starting to complete the survey, registered dietitians/nutritionists or medical doctors, and nurses, who deal with patients with nutrition-related diseases, own a smartphone and are able to understand English. Participants were excluded if they were undergraduate students of the respective disciplines (dietetics, nutrition, medicine, nursing), did not own a smartphone or were unable to understand and comply with written and verbal instructions or give informed consent.

The HCPs were recruited through (i) nutrition and/or medical conferences, workshops and congresses, (ii) dietetic and physicians associations (e-mails, monthly electronic newsletters), and (iii) social media.

### 2.1. Statistical Analysis 

We considered only completed surveys for our data analysis. The normality of data was explored with the Shapiro-Wilks Test. Descriptive statistics were used to explore the data according to current practices of HCP, app recommendation, dietary assessment (including frequency counts and cross-tabulations). A chi-square test of independence was performed to examine the relation between HCPs and their recommendation of ND apps. Multiple binary logistic regressions were used to look for statistical associations among factors (e.g., age, gender, education, profession job years) and HCPs who recommended apps. Recommendation of a ND app was the dependent variable and results were expressed as odds ratio [95% confidence interval]. All analyses were considered statistically significant at a level of *p* < 0.05. RStudio (Version 1.0.153-© 2020–2017 RStudio, Inc., Boston, MA, USA) was used. 

### 2.2. Ethics

The study was checked and declared exempt from ethics review by the Bernese Cantonal Ethics committee, Bern, Switzerland (KEK 2019-00102).

## 3. Results

**Figure 2:**
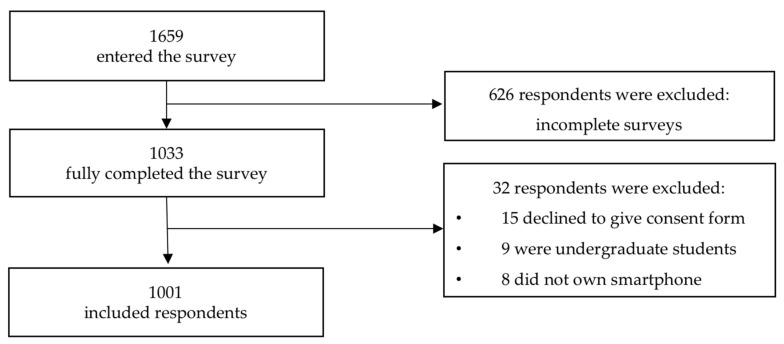
Flowchart of the participants’ inclusion.

The completion rate of the study was 62.3%. There were 1659 respondents who entered the survey and 1033 fully completed it. We finally analysed the responses of 1001 respondents who completed the survey and were also compatible with the inclusion criteria. A more detailed description of the participants’ inclusion is provided in Figure 2.

Undergraduate students of nutrition and dietetics, medicine and nursing were excluded from this study by automatically being transferred to the end of the survey once they had entered their current status.

Table 1 shows the characteristics of the respondents. Our data consist of responses from 6 continents and 73 countries.

Both the countries and the different professions of the pilot-users were represented in the survey, More specifically, the answers of the HCP in the survey in terms of professions were: Dietitian: 83.2%, MD: 7.5%, Nurse: 6.2%, Other: 3.1% The respective percentages in pilot-testers were: Dietitians: 69% (*n* = 9), MD: 15% (*n* = 2), Nurse: 7.7% (*n* = 1), Other: 7.7% *(n* = 1). Moreover: 42% (*n* = 420) of the HCP who responded to the survey came from the countries that the pilot testers came from. 

### 3.1. Current Assessment of Dietary Food Intake

In this subsection, we report the gathered information on whether the HCPs assess their patients/clients′ food intake. If so, they were asked to answer questions about which dietary assessment methods they used, the frequency of usage, as well as about the food composition database and the type(s) of technical solution used. The questions and answers are provided in Table 2.

### 3.2. Recommendation of “Nutrition & Diet” Apps

As to whether or not the HCPs recommended an app to their clients, 454 (45.4%) answered in the affirmative. In detail, 58.1% of the nurses, 45.9% of the dietitians, 37.3% of the medical doctors and 25.8% of other professions have recommended an app. HCPs were more likely to recommend an ND app if they worked in North America (*n* = 164) or Oceania (*n* = 66) rather than in Europe (*n* = 173, *p* < 0.001). Only one out of three Europeans would recommend an app, in comparison to two out of three HCPs in Oceania and North America. The probability that a female HCP (*n* = 404) recommended an ND app was higher than for a male HCP (*n* = 49, *p* < 0.001). Furthermore, there is a significant dependency between profession and recommendation (*p* = 0.01). When searching for inter-profession differences, a nurse was more likely to recommend an app than not, whereas a dietitian and a medical doctor were more likely not to recommend an app than recommend it.

In general, the probability of not recommending an app was slightly larger than of recommending it (*p* = 0.03), but did not depend on the highest level of education. Additionally, HCPs who currently use a food diary (*n* = 512, *p* < 0.001), interview (*n* = 533, *p* < 0.001) or photos (*n* = 207, *p* < 0.001) as dietary assessment method, would more likely recommend an app.

According to the logistic regression model adjusted for age, gender, education, profession and continents, job experience is a negative factor; the more experienced HCPs (in job years) were, the less likely they were to recommend an app [0.96 (0.94, 0.98), *p* = 0.04]. However, when the model was adjusted only for HCP working in the inpatient setting and profession, the HCPs from the “other” category, (*n* = 31), were less likely to recommend an app even after adjusting for all the aforementioned parameters [odds ratio = 0.403 (0.167, 0.876), *p* = 0.03].

#### 3.2.1. Reasons for not Being Satisfied with the Already Recommended “Nutrition and Diet” Apps

Of those who have recommended an app, almost one quarter (23.8%) were not satisfied with the app that they used. Respondents were asked to provide reasons for their dissatisfaction (*n* = 113) which were grouped into the following 7 themes: accuracy, Western diets-non-local databases, misunderstanding, too much interaction (manual entries), no inclusion of micronutrient content estimations, focus on weight loss rather than behavioural changes, limited access to information technology. There were 10 reasons that could not be grouped into the aforementioned themes, and a further 13 reasons that could not be used for drawing a conclusion “e.g., Because I did not” were discarded. In more detail and according to the comments, existing apps have the following disadvantages:

They focus on weight loss instead of assessing nutritional status and encouraging behavioural change;
They are inaccurate in terms of nutrient content;
They lack the analysis of micronutrient information;
They require too much interaction to add a single meal;
They do not involve the patients in their everyday use;
They provide limited access to information—so the patients usually misunderstand some nutrition facts or prescriptions;
They are designed for Western diets and many foods items are not adapted to eating patterns and needs of nonwestern countries.

#### 3.2.2. Reasons for not Having Recommended a “Nutrition and Diet” App

Of those HCPs who had not yet recommended an app (*n* = 547, 54.7%), 27.6% pointed out that this was because they did not trust the apps, 22.5% did not know of their existence and 17.6% preferred pencil and paper methods. Furthermore, 10.6% believed that using ND apps was time-consuming, 2.4% were not familiar with smartphones, 14.4% had no opinion and 30.3% had other reasons. The underlying reasons appear to be as follows:

Language/cultural aspects: (e.g., The apps are not adapted to African-Asian foods/cuisine and are not translated to countries′ spoken languages);
Literacy issues; Some HCPs work with people who have low income/socioeconomic status, low health literacy, no smartphones, live in rural areas or without internet connection;
Their job role (e.g., people working in Enteral Nutrition, Parenteral Nutrition units);
HCPs who are not allowed by their institution’s regulations to recommend apps;
The need for human interaction;
The patients′ age group (e.g., babies/children or the elderly) or the health condition (e.g., mental retardation);
Patients with eating disorders (ED) cases; this is “fuel to the fire” and thus the app is not appropriate for those with ED;
The credibility, validity, reliability of the apps;
Patients are uninterested or already have their own app.

Furthermore 76.2% of those who have not yet recommended an ND app would be willing to do so in the future.

### 3.3. Criteria, Importance of Specific Features, and Barriers for Selecting a “Nutrition and Diet” App

#### 3.3.1. Criteria

**Figure 3:**
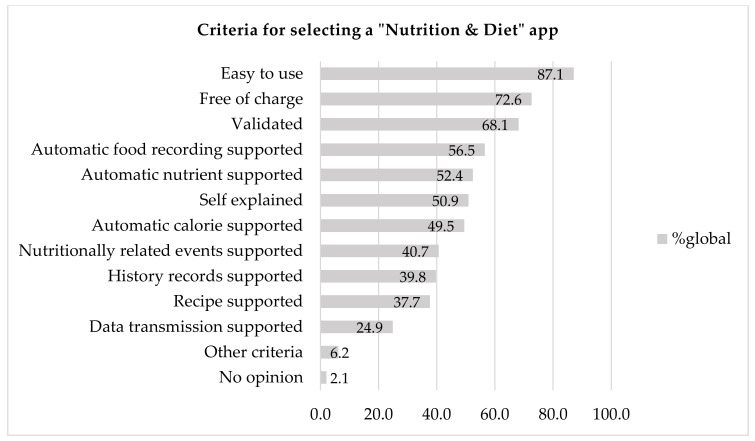
Criteria for selecting a “Nutrition and Diet” app.

The most prominent criteria for selecting an application are shown in Figure 3, and they were ease of use (87.1%), whether the app is free of charge (72.6%) and validation (68.1%). Around half of the respondents stated that important criteria are that the app supports automatic food recording (56.5%), automatic nutrient estimation (52.4%) and automatic calorie estimation (49.5%) and includes instructions for use (50.9%). 

HCPs who expressed other opinions (*n* = 62) stated that their criteria for selecting an app included recommendation by other health professionals. Other HCPs considered that it would be useful to have the option to record emotional state, mood and also appetite (hunger, satiety). Moreover, it would be helpful to have suggestions on food choices and food alternatives according to the region. Other respondents pointed out that it would sometimes be useful to be able to turn off any numerical value and to be able to display it with methods such as the plate method. This would be particularly useful for patients with ED. Lastly, the option of adding medication and nutrition supplements would be important for some HCPs. 

#### 3.3.2. Importance of Specific Features

Additionally, the participants were asked to rate the importance of the following features for an ND app, using a scale from 1 = Not important at all, 5 = Extremely important. We have split the answers into four categories, namely; basic features, automatic vs. manual features, data sharing and personalisation. History records refer to the consumed or about to be consumed meals’ saved data (photos, nutrient and energy estimations) in the smartphone that HCP can access and thus be able to retrospectively evaluate a patient′s/client′s eating and drinking history.

**Figure 4:**
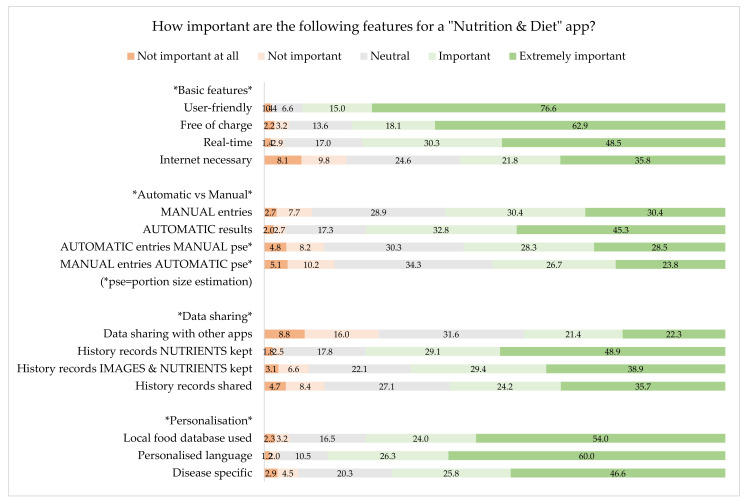
Importance of specific features for “Nutrition and Diet” apps.

As shown in Figure 4, the most important basic features are user-friendliness and also whether the app is free of charge. Moreover, the respondents prefer automatic results rather than manual ones. It seems that data sharing with other apps is not a particularly important factor influencing the HCP’s decision to recommend an app, but they would value storage of the history records of nutrients. Personalisation in terms of language (and units) and the use of local food database are important for the HCP.

#### 3.3.3. Barriers

**Figure 5:**
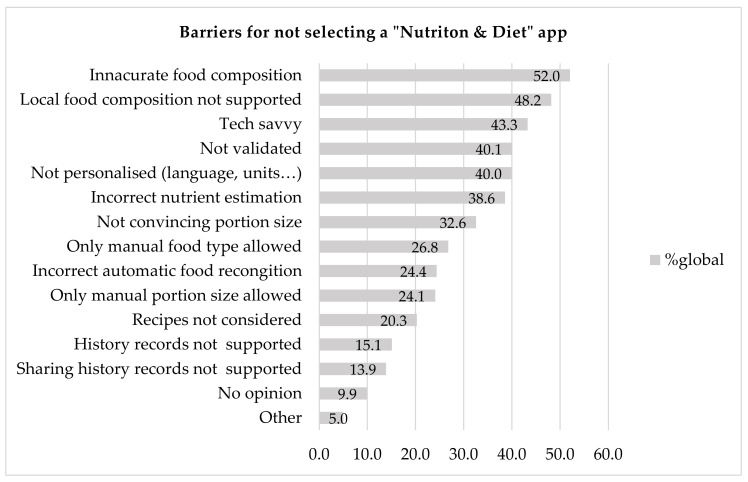
Barriers for not selecting “Nutrition and Diet” apps.

The most commonly identified barriers to ND apps, as shown in Figure 5, were the use of inaccurate databases for food composition (52%), the fact that local food composition is not supported (48.2%) and the fact that the user needs to possess technical expertise (43.3%). Moreover, nonpersonalisation in terms of spoken language or measurement units used in the app (e.g., ml or oz) and incorrect nutrient estimation are also considerable barriers. The lack of history records is not a major problem, as it was mentioned by relatively few respondents (15.1%). The same applies to the possibility of sharing history records (13.9%).

For the respondents who selected the “other” option, the main problems were that current apps focus too much on specific nutrients and calorie counting and not on health behaviours. Most of the HCPs (56%) mentioned that the existing apps actually encourage disordered eating and pose a risk of developing ED.

We sought to identify relationships between the continents and the three most important barriers. Our results showed that there is no significant relationship for the barrier of inaccurate food databases (*p* = 0.535). However, there is relationship with the other two barriers, namely lack of support for a local food database (*p* < 0.001) and technical expertise (*p* < 0.001). 

We performed analysis by logistic regression of these barriers against the continent in which each HCP lives and works. Significant relationships were verified with local food database and technical expertise. More specifically, the majority of HCPs who live in Africa stated that not having a local food database integrated in an ND app is really important [odds ratio = 0.35 (0.21, 0.569, *p* < 0.001)]. On the contrary the majority of HCPs who live in North America [odds ratio = 6.297(3.689, 11.0711), *p* < 0.001] and Oceania [odds ratio = 3.246 (1.77, 6.0723), *p* < 0.001] would not consider it important. When it comes to technical expertise, HCPs who live in Europe would not find it an important barrier [odds ratio = 2.227(1.4, 3.55), *p* < 0.001]. On the other hand, about an equal number of HCPs from South America would consider this barrier important or not [odds ratio = 4.7(2.022, 12.019), *p* < 0.001].

### 3.4. Preferences for the Display of the Results on the Screen

#### 3.4.1. Macronutrients

HCPs were asked questions on how they wished to have the results for nutrients displayed on the screen (type, visualisation of results and amount of nutrients). The respondents were asked how important they thought it was that the results for energy, carbohydrates, protein and fat results were presented in the ND app. When respondents were asked to rate this (using a scale of 1 = Not important at all, 5 = Extremely important), about two thirds (66.7%) of the respondents considered that this was extremely important for energy, 74% for carbohydrates, 69.2% for protein and 69.2% for fat estimation. Less than half of the respondents (45.2%) considered that it was extremely important to display the micronutrients. 

HCPs were also asked how they would prefer the results of macronutrients to be displayed on the ND app’s screen: whether they would prefer to see their exact values or the exact values combined with a traffic light system. For energy, most respondents preferred the accurate value in kcals. The respondents were also asked how they would prefer the size of the meal to be displayed on their screen and 55.3% (*n* = 554) preferred household measures (e.g., cups, spoons, etc.), 30.1% (*n* = 301) standard units (e.g., grams, ml, etc.), 11.4% (*n* = 114) portion size (small, medium, large) and 3.2% (*n* = 32) would choose something else.

#### 3.4.2. Accuracy in Relation to Time Needed to Obtain a Result

In terms of preferences related to the accuracy of the estimations and time needed to output the results, we provided the following three options: i) the user needs to capture two images, from specific angles, which are then used for the volume estimation of the food. From the moment of the second image capturing the results are available within 5 seconds with estimation accuracy most probably as high as that of a dietitian, ii) the user needs to capture one image, which is used for the volume estimation. The results are available in real-time, with an estimation accuracy most probably lower than that of a dietitian, and iii) the user needs to record a short video, which is used for the volume estimation. The results are available after 5 seconds from the moment the video stopped recording, with an estimation accuracy most probably higher than that of a dietitian. From these, 59.9% (*n* = 600) opted for the first method, 20.8% (*n* = 208), would prefer the second faster, but less accurate method and 19.3% (*n* = 193) chose the highest accuracy possible, offered by the third method.

## 4. Discussion

To the best of our knowledge, this study is the largest global study of HCPs that addresses their recommendation of ND apps, as well as their perceptions, preferences and barriers towards their recommendation. Overall, 45.4% (*n* = 454) of the participants had recommended an app to their clients/patients. This percentage is comparable to a recent study [17] that surveyed Canadian dietitians and found that 40.5% of respondents had recommended diet apps to clients. In another study, 84% of the survey’s dietitians had recommended a nutrition-related app to their patients [15]. Moreover, 56% of the clinicians who participated in a similar study (*n* = 398) used the app for working purposes [14]. Similarly, 83% of the responders (nutrition and dietetics practitioners) (*n* = 62) of another survey, recommended ND apps to patients/clients [18]. In another study, 57/176 (32.4%) sports dietitians used ND apps in tracking and assessment of their athletes’ intake [16]. We suppose that the recommendation rate of 45% in our study may stem from socioeconomic differences that arise from the plethora of participating countries coming from six different continents.

In our study 42.3% of the respondents aged between 21 and 30 years old, which is the dominant age range. The predominant age range in similar studies were: 30–39 (34%) [16], 26–35(44%) in [15], 25–34 (51.7%) in [17], 30–39 (45.1%) in [22] and 50–59 (32%) in [14], while Sauceda et al. [18] did not report the age of participants. Therefore, most similar studies have a similar or slightly older predominant age range than our study, while in Karduck et al. the respondents were even older [14]. It is challenging to conclude specific parameters that might drive the aforementioned differences since no relative justification is provided in the studies.

Regarding the gender distribution, in our study 84.7% were female HCPs. This is expected due to the inherent over-representation of females in the dietitian’s domain which constitutes 83.2% of the US [23] and 94.6% of the Australia dietitians [24]. In similar studies where only dietitians were included, the respective percentage was 95% [15], 98.3% [17] and 96.1% in [14]. In Zhang et al. [22] the participants were diabetes specialists and the respective percentage was 67.6%. 

We found that HCPs most frequently use the following arguments for not selecting such an app: the displayed food compositions are inaccurate; local food composition is not supported; people who use the app need to possess a certain level of technical expertise; the app is not personalised in terms of language/units; the nutrient estimation is incorrect. Limitations of ND apps which were mentioned in Jospe et al. [16] are in agreement with our study. More specifically, of sports dietitians (*n* = 22/54), 41% pointed out that the most important barriers were “the inaccurate nutrient database is inaccurate, missing foods in it while being not country-specific”. It was also mentioned (*n* = 22/54) by 22% that incorrect portion size selection as well as incorrect food selection (*n* = 7/54),13% were also considerable limitations. However, from the 176 participants in the study only 54 provided their opinion on criteria and barriers. Doubts about the accuracy of diet apps were also expressed in another study, in which the main limitations were expressed to be the accuracy of the results output, poor quality and poor usability [17]. In the same study, concerns about privacy protection, inaccurate energy calculations, and also the fact that apps focused mainly on calorie estimation rather than healthy eating were also expressed [17]. In another study, the HCPs considered that health apps were useful in their everyday practice. Nevertheless, several limitations related to patient use were found; lack of user-friendliness, lack of appropriate use for certain populations (e.g., the elderly), problems with patient access (e.g., population without smartphones), and time commitment needed [14]. An additional obstacle was the lack of awareness of the best apps to recommend (41%) [15]. In the same study, the researchers also mentioned that important barriers were no access to smart devices (51%) and also a lack of necessary infrastructure, such as no Wi-fi access.

In our study, a HCP would employ the following criteria when selecting an ND app: Ease of use, whether the app is free of charge, validation, support of automatic food recording and nutrients estimation. In [17] the factors that affected dietitians’ use of apps and their recommendations to clients included accessibility, content quality, usability, compatibility, cost, knowledge, interest, suitability, willingness, ability to pay, and ability to use apps at work. In [16], sports dietitians (54 out of the 176) answered that benefits of ND apps are that they are ubiquitous (*n* = 27), 50%, convenient (*n* = 14), 26% and easy to use (*n* = 12), 22% [16].

In our study, 76.2% of the HCPs who had not yet recommended an ND app to their patients/clients were willing to do so in the future. Similarly, in another study, 83.6% of respondents who did not use apps in practice were interested in future use [17]. Additionally, an interesting finding is that 22.5% of our respondents, who had not yet recommended an app, were not aware of the existence of diabetes apps. Similarly, in a study conducted among Chinese diabetes specialists, 34.9% (212/608) did not know of the existence of diabetes apps [22].

There were 626 respondents who started the study questionnaire, but did not complete it fully. Possible reasons for this high number of incomplete survey responses include problems with internet connection, disruption of unexpected events or even loss of interest or other tasks of higher priority. Even though it would be interesting to know, we did not have a way to check why someone did not participate in our study considering the way it was disseminated. Possible reasons include low interest in technological development or use of applications in their clinical practice.

The main strength of the study is that it includes the opinions of HCPs from around the world, so it is one of the broadest studies of its kind on this topic. Moreover, efforts were made to eliminate possible biases related to the investigators’ individual interests. To this end, the team who designed the study was multidisciplinary, thus incorporating perspectives from different fields and eliminating biased reporting. The survey’s distribution was wide with a variety of sources (conferences, organisations etc.). Furthermore, we did not limit the questioning to a single environment, such as hospitals or conference participants and we investigated app integration into HCP practice in various settings. The limitations include the possible over-representation of younger HCPs, due to their familiarity and use of social media, albeit with substantial job experience. As a result, the perspectives of older HCPs are not well represented, so we could not generalise the results across all ages and levels of experience. Additionally, the convenience sampling adopted may not underline entirely the probabilistic distribution of the HCP population. Another limitation is that the survey was restricted to HCPs who had access to the internet. Non-English speakers are not represented, since the survey was designed and distributed only in English. 

Future studies could explore the responses of end users to similar questions and then to compare their answers to those of HCPs—perhaps there is common ground. Studies that include more men and more medical doctors and nurses may be needed, in order to gain a clear overview of their preferences and to be able to generalise the results. Future research must evaluate the integration of the apps into everyday clinical practice. Follow up studies, focusing on gathering more data from developing countries would also be of vital importance. More validated apps are needed, as well as research to define how and when apps may most effectively support their work. The co-ordination between HCPs, AI experts and computer scientists is important—not only in the initial planning of a dietary assessment/monitoring app, but also in all the stages of its development. Training with validated apps should be provided by professional associations and this should not only focus on the commercially available apps, but also on how these apps could be integrated into their everyday practice.

## 5. Conclusions

This study investigated the perspectives, acceptability and preferences of HCPs who work with nutrition-related diseases. We found that slightly less than half of the surveyed HCPs recommended ND apps to their patients/clients. Therefore, there is room for improvement and broader adoption of ND apps to dietetic practice as mHealth research is rapidly developing. Future research should continue to address HCPs who cope with nutrition-related diseases and should study how apps are best used in dietary monitoring and assessment. Lastly, attention should be given to the criteria for recommending an app and should emphasise the preference on validated, user-friendly, free of charge apps incorporating local food databases.

## Figures and Tables

**Table 1:** Demographic characteristics of the respondents (*n* = 1001).

(*n* = 1001)		%	*N*
Sex	Female	84.70%	848
	Male	15%	150
	Neither/prefer not to disclose	0.30%	3
Age average (years, SD)	34.4 (10.2)		
Age range	21–30	43.20%	433
	30–39	30.80%	308
	40–49	16.40%	164
	50–59	8%	80
	60–69	1.20%	12
	>70	0.40%	4
Job experience average (years, SD)	7.7 (8.2)		
Continent	Europe (29)	44.50%	446
(number of countries)	North America (4)	24.80%	248
	Oceania (2)	10.40%	104
	Africa (13)	8.70%	87
	Asia (18)	7.60%	76
	South America (7)	4%	40
Profession	Dietitian and/or Nutritionist	83.20%	833
	Medical Doctor	7.50%	75
	Nurse	6.20%	62
	Other	3.10%	31
Highest educational	BSc	37.20%	373
level	MSc	43.30%	433
	Diploma	4.10%	41
	PhD	7.70%	77
	Medical Degree	4.30%	43
	Other	3.40%	34
Diseases treated *	Weight management	73.70%	738
	Diabetes	63.50%	636
	Cardiovascular diseases	50.60%	507
	Gastrointestinal diseases	45.30%	453
	Malnutrition	40.70%	407
	Cancer	25.70%	257
	Kidney diseases	25.00%	250
	Eating disorders	23.40%	234
	Other diseases	18.90%	189
Carbohydrate (CHO)	Portions	52.30%	524
estimation technique used	CHO counting	45.30%	454
for people with diabetes*	Food labels	43.80%	438
	Apps	18.10%	181
	Weighing foods	16.90%	169
	No education	3%	30
	Other CHO education	4.40%	44
Main patients′ age group	18 to 65 years old	79%	791
	Over 65 years old	12.50%	125
	Up to 12 years old	6.40%	64
	12 to 17 years old	2.10%	22
Job setting *	Private Practice	48.50%	486
	Hospital inpatients	46%	461
	Hospital outpatients	41.80%	418
	Home for the elderly	11.70%	117
Operating System of their	iOS (Apple iPhone)	51.80%	519
own smartphone	Android	47.60%	476
	Windows	0.30%	3
	I do not know/I do not want to answer	0.20%	2
	Other	0.10%	1

* multiple answers: the respondents chose all the possible answers; the sum is not equal to 100%.

**Table 2:** Currently used dietary assessment methods.

Asked Question		Given Answers	Percentage	*N*
Do you assess your patients/clients’ food intake?	No		10.20%	102
If yes, how often?	Yes		89.80%	898
		Once/twice per month	40.10%	360
		Every few months	25.50%	229
		Once a week	21.60%	194
		Once per year	3.30%	30
		At a different frequency	9.50%	85
Which method of dietary assessment do you use? *		24-hour recall	55.20%	496
		Interview	53.30%	479
		Written food diary	51.20%	460
		FFQ	23.20%	208
		Food diary via photos	20.70%	186
		Other methods	6.60%	59
Do you keep records of your patients’ eating habits?	No		30%	300
	Yes		70%	700
Do you analyse the recorded data obtained?	No		61.20%	612
If yes, which database do you use?	Yes		38.80%	388
		USDA	32.40%	126
		Other	28.50%	111
		None	27.30%	106
		DACH	6.40%	25
		UK database	5.40%	21

* multiple answers: the respondents chose all the possible answers; the sum is not equal to 100%.
